# Supplementary material for: A continuous fish fossil record reveals key insights into adaptive radiation
Source: Nature. 2023 Oct 4;622(7982):315–20. doi: 10.1038/s41586-023-06603-6 (PMC10567567; doi:10.1038/s41586-023-06603-6)
Supplement: Supplementary file 1 — Supplementary Figs. 1–5, Tables 1–2 and References. [file 41586_2023_6603_MOESM1_ESM.pdf]

nature portfolio

https://doi.org/10.1038/s41586-023-06603-6

# Supplementary information

# A continuous fish fossil record reveals key insights into adaptive radiation

In the format provided by the authors and unedited

# Supplementary materials

## The study area

The sediment cores were collected in an offshore to inshore gradient in the Tanzanian sector of Lake Victoria, East Africa (*Suppl. Fig. 1*). The lake is large with a surface area of about 68,800 km$^2$ and shallow with a maximum depth of about 68m and an average of ~40m[1]. The cores were collected in 2018 from four sites referred to as LV1, LV2, LV3, and LV4, respectively. They were collected along a transect of increasing water depths (LV3 at 13 m, LV2 at 22 m, LV1 at 37 m, LV4 at 63 m) and distance from shore (LV3 at 2 km, LV2 at 6 km, LV1 at 9 km, LV4 at 30 km from the shore) in the Shirati Bay area (*Suppl. Fig. 1*).

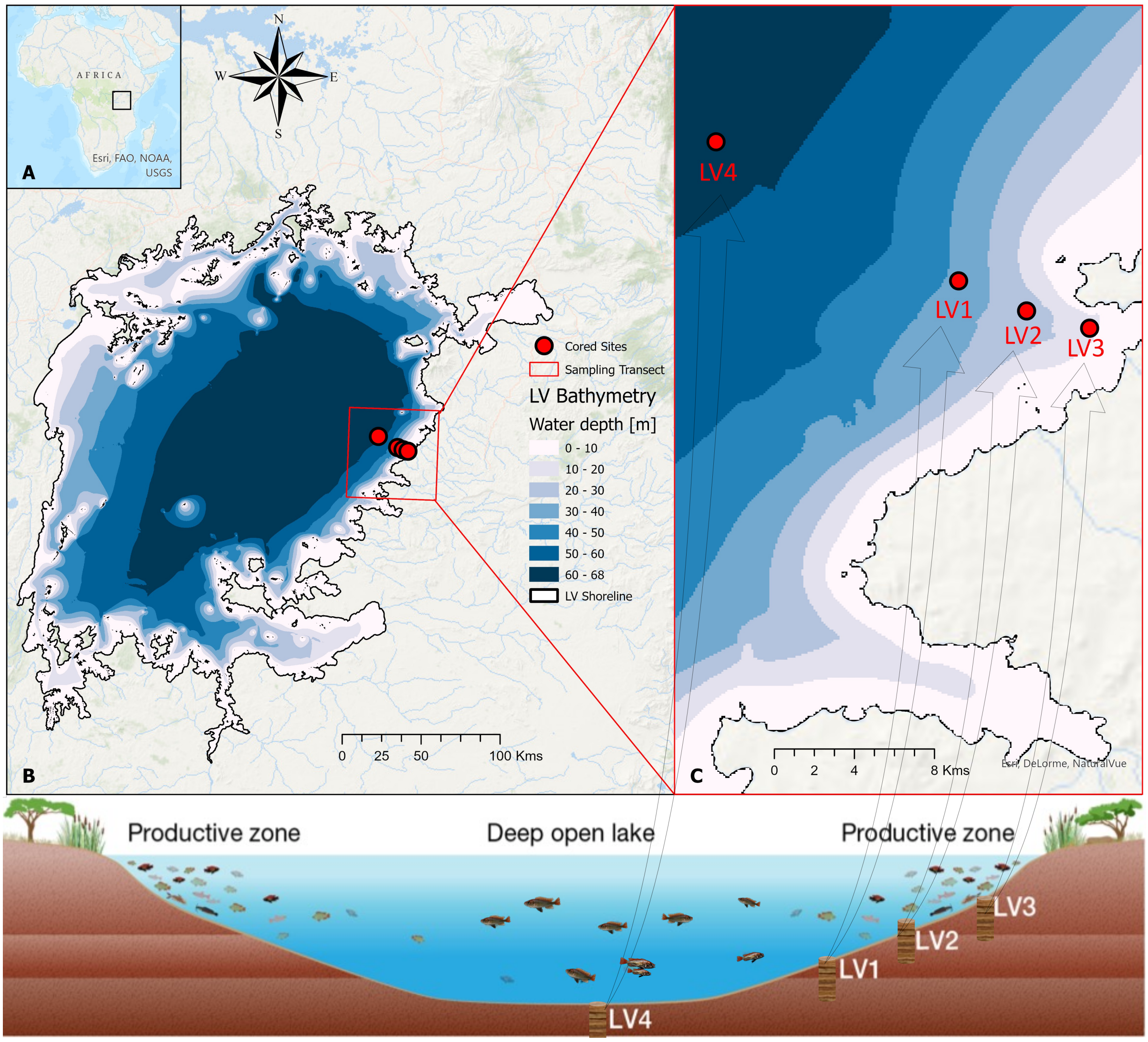


**Supplementary Figure 1**. (A) African continent map with the box showing Lake Victoria. (B) Lake Victoria map with isobath[2] of 10m increments. (C) The coring sites on the transect out of Shirati Bay from deep to shallow (LV4 at 63 m, LV1 at 37 m, LV2 at 22 m, and LV3 at 13 m) and offshore to inshore (LV4 at 30 km, LV1 at 10 km, LV2 at 6 km, and LV3 at 2 km from the shore).

# The fish fossil influx

The fossil influx was determined by initially dividing the fossil number by the sediment volume (number of fossils per cm−3) to determine the fossil concentration. Then the fossil concentrations were multiplied with the sediment accumulation rates from (cm/yr) [3] to calculate the fossil influx (number of particles cm−2 yr−1) (*Suppl. Fig. 2*).

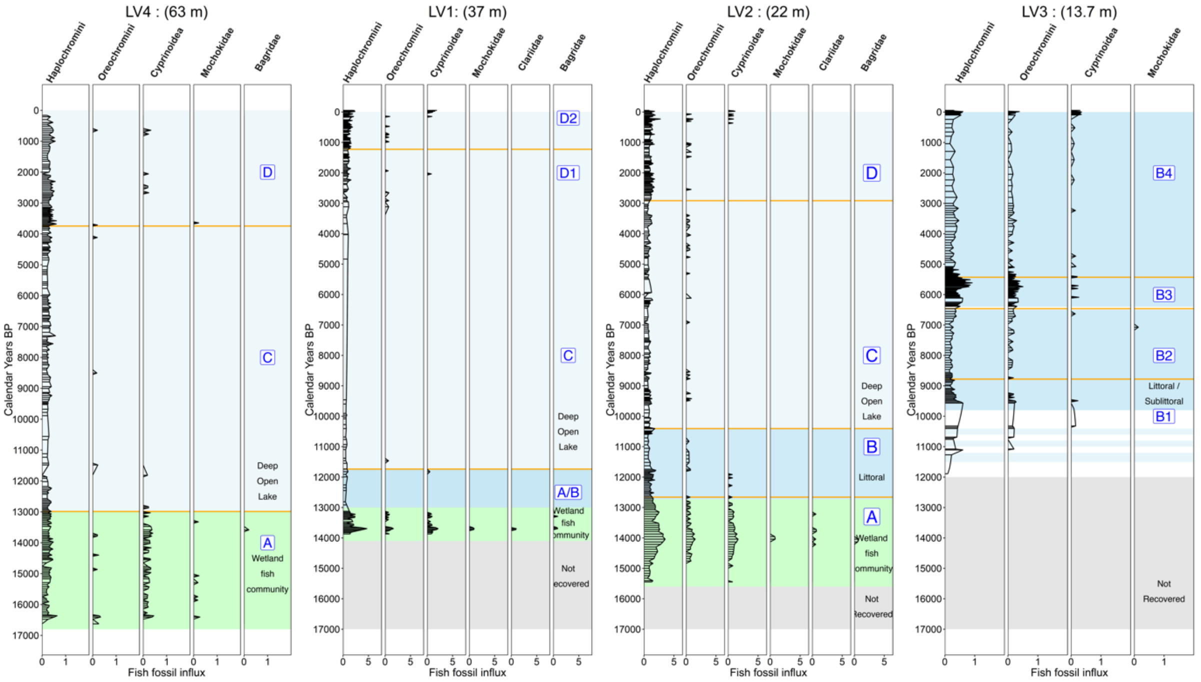


**Supplementary Figure 2**: The fish fossil influx since the origin of the modern lake from the tooth fossils per area in an onshore-offshore coring transect. The bars represent the fossil influx of the recovered fish taxa. The solid orange lines are statistically significant multivariate changepoints detected across all taxa using the E-divisive method[4]. The letters (A-D) denote different major phases or habitat stages as defined by the fish community bounded by change points and shared between the sites. Where A (green shade) represents the wetland fish community, B (dark blue) the littoral/sublittoral phase and C & D (light blue) indicate the deep open lake phases that differ in fossil abundance. The grey shade represents depth not cored and the white spaces in LV3 are intervals not screened for fossils.

## The reference catalog and fossils

A digital photograph catalog of modern tooth specimens of Lake Victoria fishes was created as a reference collection. The collection comprised 44 species representing nine families and one superfamily that formed the modern assemblage in Lake Victoria (*Suppl. Table 1*, *Suppl. Fig. 3*). Additionally, a compilation of fish fossils belonging to different fish groups discovered in the fossil record, showcasing a range of preservation conditions from the four cored sites LV4, LV1, LV2, and LV3 (*Suppl. Fig. 4*).

**Supplementary Table 1**: Fish species from Lake Victoria that we used to build a reference library of teeth which we used together with literature to assign fossils to major taxa

| | Family/Lineage | Species |
|---|---|---|
| Characids | Alestidae | *Brycinus jacksonii* |
| Catfishes | Bagridae | *Bagrus docmak* |
| | Clariidae | *Clarias sp.* |
| | Mochokidae | *Synodontis victoriae* |
| | Schilbeidae | *Schilbe mystus* |
| Carp-relatives | Cyprinoidea/Cyprinidae | *Enteromius sp.* |
| | Cyprinoidea/Cyprinidae | *Labeo victorianus* |
| | Cyprinoidea/Danionidae | *Rastrineobola argentea* |
| Lates perches | Latidae | *Lates niloticus* |
| Spiny eels | Mastacembelidae | *Mastacembelus frenatus* |
| Cichlids | Cichlidae/Oreochromini | *Oreochromis leucosticus* |
| | | *Oreochromis esculentus* |
| | | *Oreochromis variabilis* |
| | | *Oreochromis niloticus* |
| | Cichlidae/Haplochromini | *Astatoreochromis alluaudi* |
| | | *Astatotilapia nubila* |
| | | *Enterochromis paropius* |
| | | *Gaurochromis hiatus* |
| | | *Haplochromis sp “*purple yellow” |
| | | *Harpagochromis cf. serranus* |
| | | *Labrochromis stone* |
| | | *Lipochromis melanopterus* |
| | | *Lithochromis sp*. “scraper” |
| | | *Lithochromis sp. “*yellow chin” |
| | | *Mbipia lutea* |
| | | *Mbipia mbipi* |
| | | *Neochromis gigas* |
| | | *Neochromis omniceruleus* |
| | | *Neochromis rufocaudalis* |
| | | *Neochromis sp. “*uniscupid scraper” |
| | | *Paralabidochromis chilotes* |
| | | *Paralabidochromis cyaneus* |
| | | *Paralabidochromis flavus* |
| | | *Paralabidochromis sp. “*short snout scraper” |
| | | *Platytaeniodus degeni* |
| | | *Psammochromis riponianus* |
| | | *Paralabidochromis sauvagei* |
| | | *Ptyochromis xenognathus* |
| | | *Pundamilia macrocephala* |
| | | *Pundamilia nyererei* |
| | | *Pundamilia pundamilia* |
| | | *Pundamilia sp.* pink anal |
| | | *Yssichromis laparogramma* |
| | | *Yssichromis pyrrhocephalus* |

The major taxonomic groups of fishes in the Lake Victoria catchment, their habitat affiliations and tooth traits:

Alestidae

Two species of Alestidae have been recorded in Lake Victoria (*Brycinus sadleri,* and *Brycinus jacksonii*)[5]. They are found in shallow inshore areas with aquatic vegetation and papyrus[6]. The teeth of alestids are tri- to multi-cuspidate as adults and very irregularly shaped with the neck hardly differentiated against the crown[7] (*Suppl. Fig. 3*).

Bagridae

This family has one species occurring in Lake Victoria (*Bagrus docmak*). They are found in all parts of LV at variable depths but are confined to shallow rocky areas for breeding and nursery[8]. Their teeth are long unicuspid, pointy, and sometimes hook-like (*Suppl. Fig. 3*).

Clariidae

There are six species of clariids occurring in Lake Victoria. They are found in shallow wetland habitats (*Clarias liocephalus*) to offshore deep water but all of them, including the little-known deepwater species (*Xenoclarias eupogon*), seem confined to inshore zones for breeding[9], including marginal waterlily and papyrus swamps (*C. gariepinus*), and rocky shores (*C. gariepinus, X. eupogon*)[10]. The teeth of clariids are unicuspid, pointy and thin[11] (*Suppl. Fig. 3*).

Mochokidae

This family of African catfishes has two species occurring in Lake Victoria (*Synodontis afrofischeri and Synodontis victoriae*). *Synodontis victoriae* occurs from shallow to deeper waters but migrate into rivers to spawn, while *Synodontis afrofischeri* prefers waters less than 20m deep[12]. The teeth are chisel-shaped, elongated and often bent with a characteristic hook at the tip[8] (*Suppl. Fig. 3*).

Schilbeidae

The African butter catfish has one species (*Schilbe intermedius*) occurring in Lake Victoria. They inhabit inshore areas and live pelagic, but migrate up rivers for spawning[6]. Their teeth are small and unicuspid with a sharp point[13] (*Suppl. Fig. 3*). They resemble haplochromines in length-to-width ratio but are pointier.

Cyprinoidea

The “carp-like” fish superfamily has 17 species in Lake Victoria belonging to two families: Danionidae (only *Rastrineobola argentea*) and Cyprinidae (all other species). In LV, they are found in a variety of habitats but most species are confined to swamps, streams, and inshore waters and the larger species migrate to rivers to spawn (genera *Labeo* and *Labeobarbus*). In the main lake weed beds (various species in the genus *Enteromius*), rocky shores (*Labeo*) and very shallow exposed rocky habitats (genus *Garra*) are the main habitats. Two species live in the pelagic (*Rastrineobola argentea* and *Enteromius profundus*)[8]. Cypriniforms have distinctive pharyngeal teeth with hooked cusps (*Suppl. Fig. 3*), usually not more than three rows [14,15].

Latiidae

One species of the Lates perches family (*Lates niloticus*) was introduced to Lake Victoria in the 1950s[16]. They inhabit both shallow and deep waters[17]. The teeth are unicuspid and pointy but short with a very broad base (*Suppl. Fig. 3*).

Mastacembelidae

The spiny eels have one species occurring in Lake Victoria (*Caecomastacembelus frenatus*). They are found in marginal swamps, streams and shallow rocky reefs. They have numerous small unicuspid and pointed teeth[8,18] (*Suppl. Fig. 3*).

Cichlidae: Oreochromini

The lineage of cichlid fish with four species occurring in Lake Victoria (two introduced in the 1950s, *Oreochromis leucosticus* and *Oreochromis niloticus* and two natives *Oreochromis variabilis* and *Oreochromis esculentus*). They prefer inshore waters, with a maximum of 15m water depth[19]. *Oreochromis* have bicuspid and tricuspid oral teeth with a very tall and slender neck and a large crown with distinctively shovel-shaped major cusp with a very large flange and a small minor cusp and they also have long and slender pharyngeal teeth[20] (*Suppl. Fig. 3*).

Cichlidae: Haplochromini

The most diverse lineage in Lake Victoria with over 500 species. They are found in all habitats[21,22]. The outer oral jaw dentition of different species varies from bicuspid (the most widespread condition) to unicuspid (only in adults of some trophic specialists) or tricuspid (very rare), with varying shape and size. Haplochromines have small tricuspid teeth in the inner oral jaw tooth rows. Their pharyngeal teeth vary from thin and highly asymmetric and oblique to hooked to heavy molariform, always with a major cusp and a much smaller minor cusp (*Suppl. Fig. 3*).

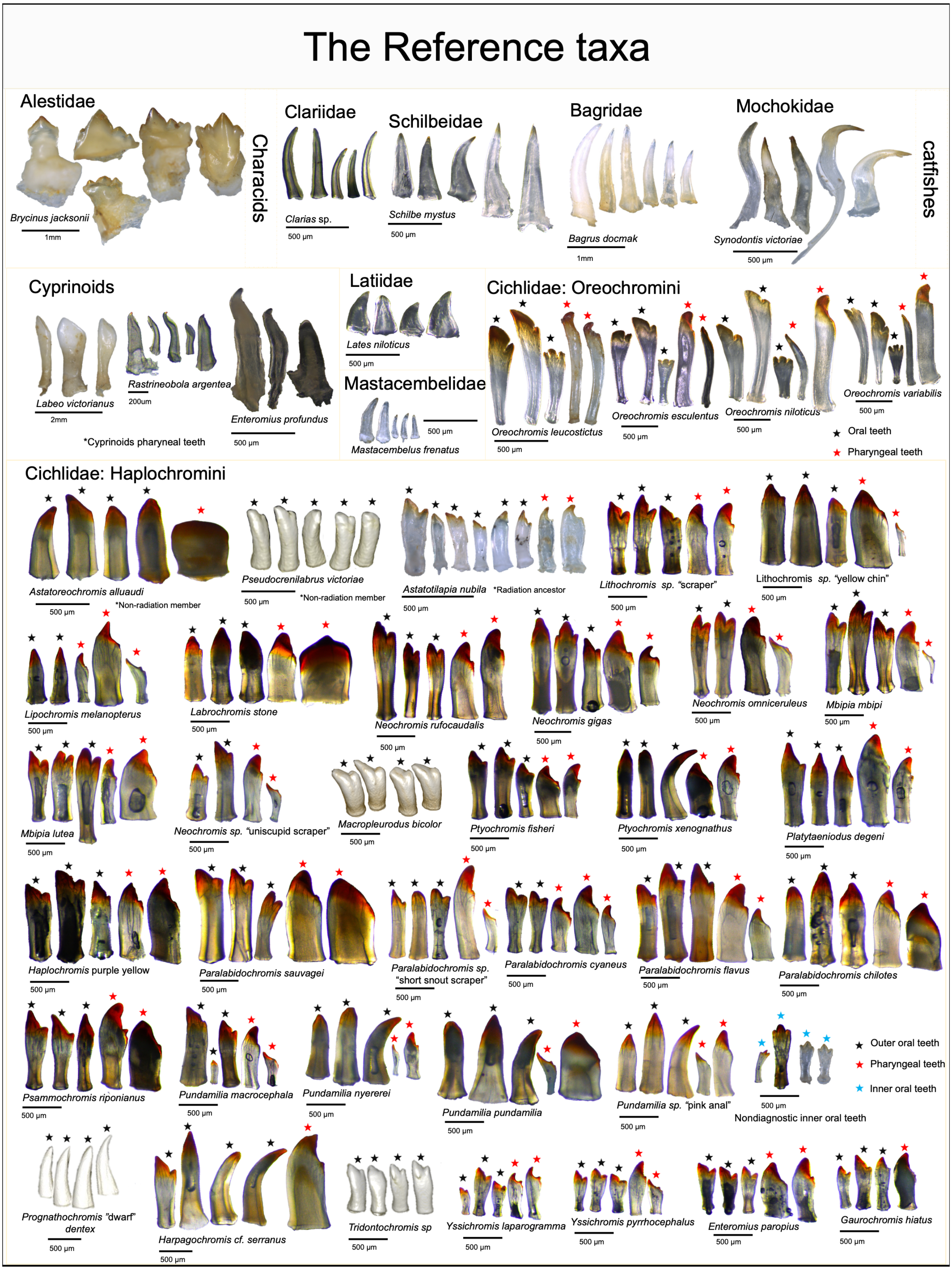


**Supplementary Figure 3**: Examples of representative teeth from various modern fish taxa of Lake Victoria.

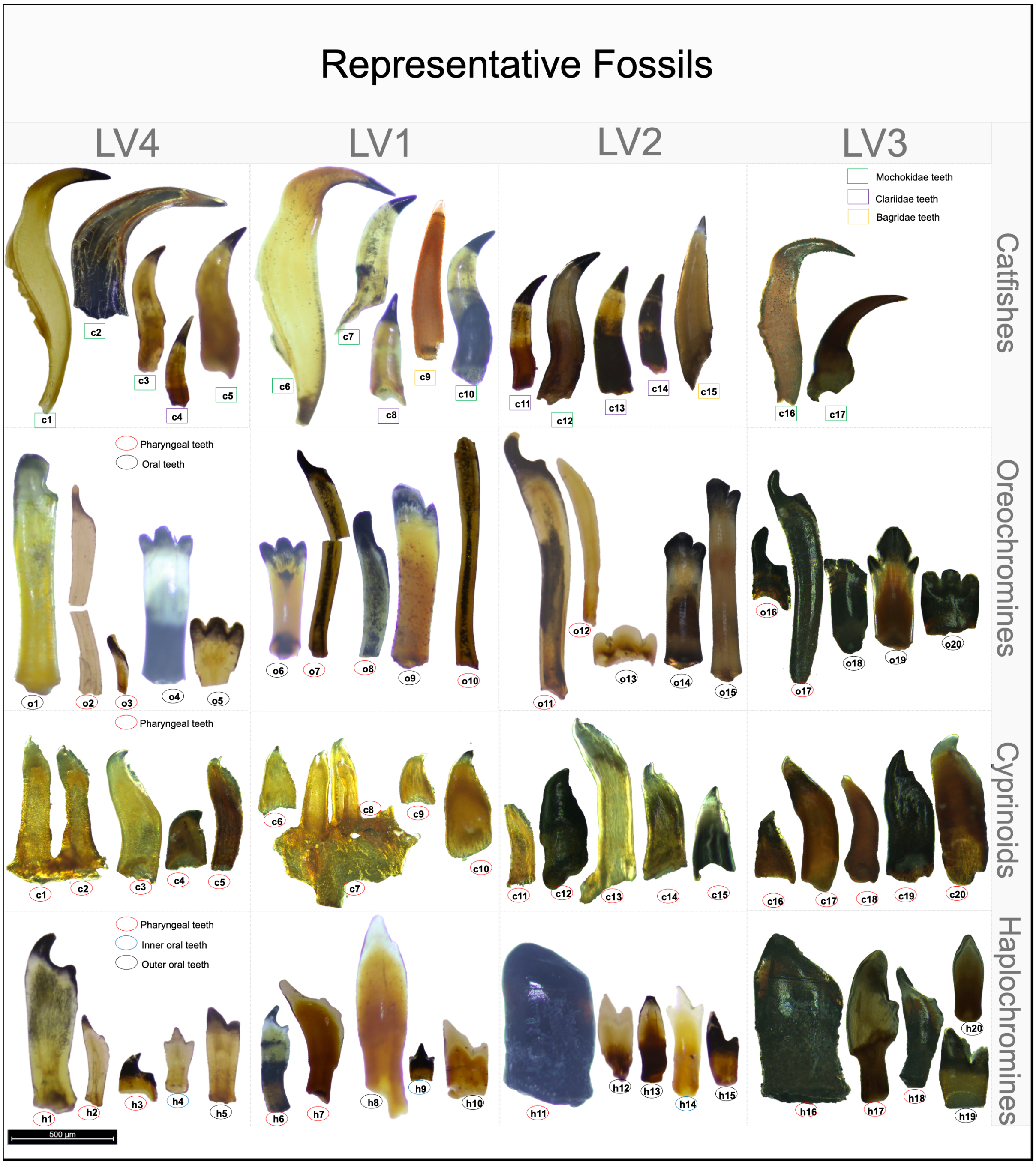


**Supplementary Figure 4**: The fish fossils. The Haplochromini group denoted as h1-h20 (red circled are pharyngeal teeth, blue circled are inner oral teeth, and black circled are outer oral teeth), Cyprinoidea as c1-c20 (red circled are pharyngeal teeth), Oreochromini teeth as o1-o20 (red circled are pharyngeal teeth, and black circled are oral teeth), and the catfishes as c1-c17 (The green square are Mochokidae, purple square are Clariidae, and yellow square are Bagridae), across the four locations.

**Supplementary Table 2**: The total number of fish tooth fossils recovered from the sediment cores per site and the percentage assigned to each family with general habitat preferences of families

| | | LV4 | LV1 | LV2 | LV3 | LV-Total | Habitat Affiliations |
|---|---|---|---|---|---|---|---|
| | Fossils counts | 931 | 1368 | 2842 | 2481 | 7623 | |
| Fish taxa % | Bagridae | 0.11 | 0.37 | 0.11 | 0 | 0.12 | Rivers and lakes from shallow to deep but confined to rocky substrate for breeding |
| | Clariidae | 0 | 0.37 | 0.18 | 0 | 0.13 | The species are widespread across water depths but most common in inshore waters including in the marginal waterlily and papyrus swamps. Even the species in deep water depend on shallow rocky shores for breeding |
| | Mochokidae | 0.75 | 0.37 | 0.18 | 0.04 | 0.24 | Rivers and lakes from shallow to deeper lake waters but confined to shallow habitat for breeding or migrating up rivers |
| | Cyprinoidea | 10.7 | 6.22 | 4.61 | 1.23 | 4.53 | Genus *Enteromius*: inshore lake habitats, swamps and rivers<br>Genus *Garra*: uppermost littoral zone (top 1-2m) on exposed rocky shores<br>Genus *Labeo*: over inshore rocky substrates, migrates up rivers to spawn<br>Genus *Labeobarbus*: inshore waters and rivers, migrates up rivers to spawn<br>Genus *Rastrineobola*: in- and offshore pelagic zone |
| | Haplochromini | 85.3 | 89.5 | 89.3 | 70.9 | 82.8 | The radiation ancestor *Astatotilapia nubila*: swamps and shallow waters<br>The radiation members: widespread across all habitats |
| | Oreochromini | 1.29 | 2.71 | 4.15 | 8.23 | 4.90 | *Oreochromis esculentus*: up to 15m water depth<br>*Oreochromis variabilis*: shallow zones of rocky reefs, vegetated lake shore and lagoons |
| | Unknown | 1.83 | 0.44 | 1.51 | 19.6 | 7.33 | |

## The direction of change between different phases

The boxplots were used to summarize each taxon's mean relative abundance for each habitat phase. A two-sided t-test was computed to infer significant differences between phases. The difference in relative fish abundances between change point delineated habitat phases along the offshore deep (left) to inshore shallow (right) coring transect. The LV4 site phases (*Suppl. Fig. 5a*); were A (n=56), B (n=30), C (n=99), and D (n=70). The LV1 site phases (*Suppl. Fig. 5b*); A/B (n=30), C (n=33), D1 (n=45), and D2 (n=30). For LV2 phases (*Suppl. Fig. 5c*); A (n=40), B (n=38), C (n=121), D1 (n=30), D2 (n=34) and D3 (n=30). LV3 site remained throughout in the littoral/sublittoral phases B1 (n=30), B2 (n=30), B3 (n=37), and B4 (n=57) (*Suppl. Fig. 5d*). Each plot shows the mean relative abundances of each major fish taxon for each statistically delineated habitat phase and vertical lines indicating significance differences between phases using two-sided t-tests (*Suppl. Fig. 5a-d*). Asterisks indicate the statistical significance (ns means no significant change, * Pvalue $\leq 0.05$, ** Pvalue $\leq 0.01$, *** Pvalue $\leq 0.001$, and **** $P \leq 0.0001$) of differences between phases for each taxon. The center line of the boxplot shows the median, the red dot shows the standard error mean, the upper box bound is the 75th percentile and the lower box bound is the 25th percentile. The grey dots show the observations outside the 25th and 75th percentile. The lower-end line whisker shows the minimum and the upper-end line whisker the maximum observed value.

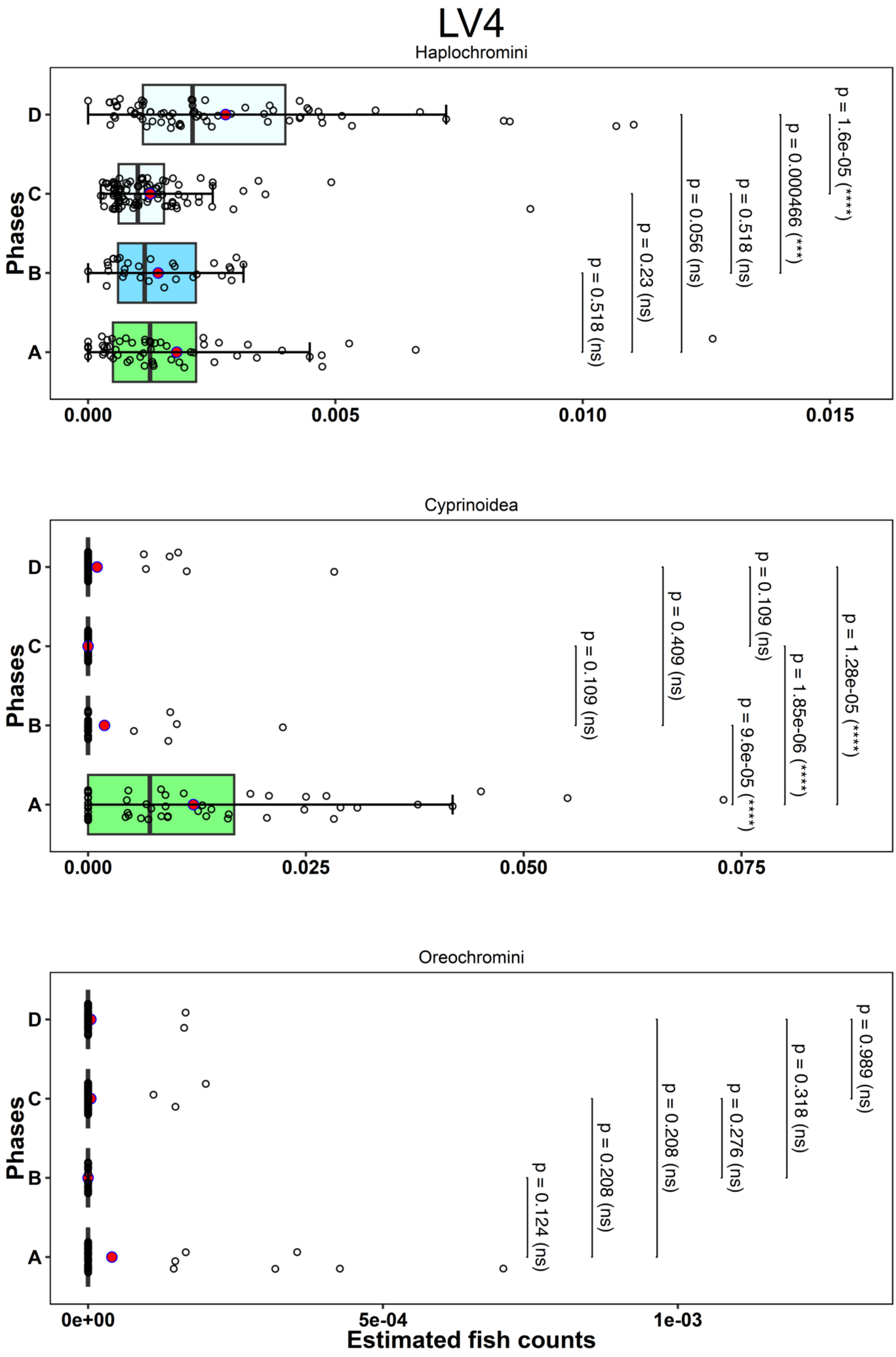


**Supplementary Figure 5a**: Boxplot from the most offshore site, LV4.

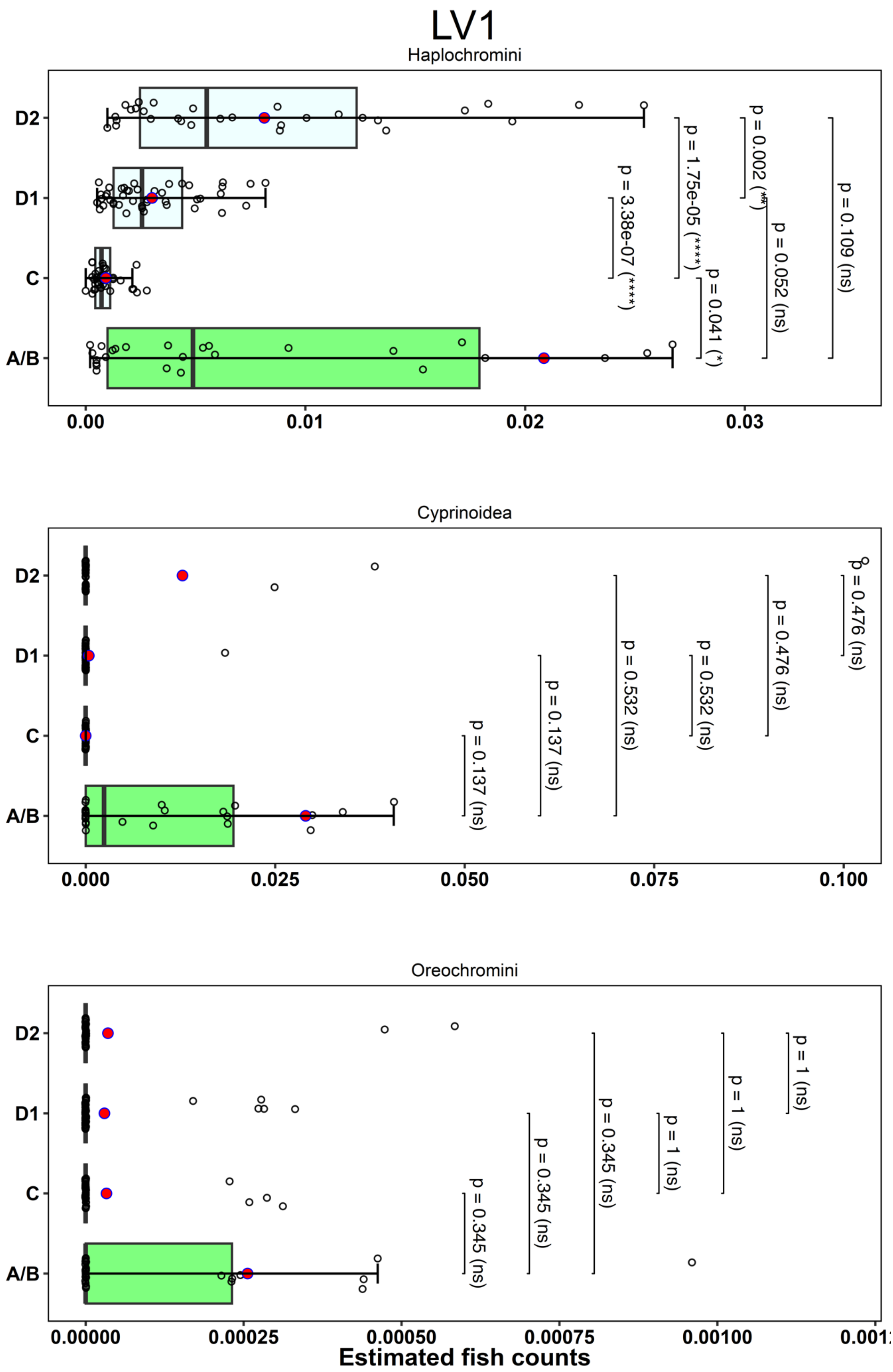


**Supplementary Figure 5b**: Boxplot from the most offshore site, LV1.

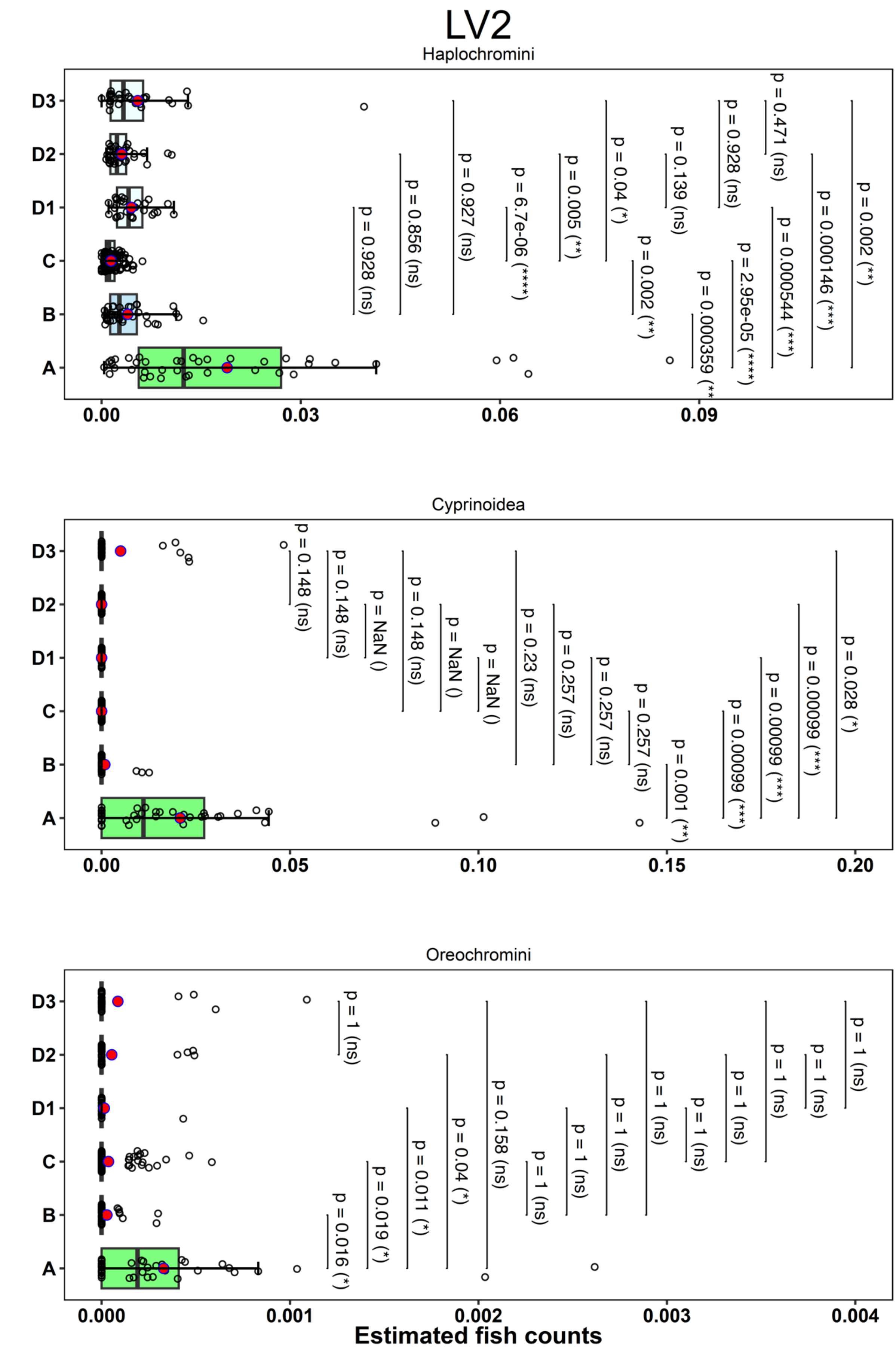


**Supplementary Figure 5c**: Boxplot from the most offshore site, LV2.

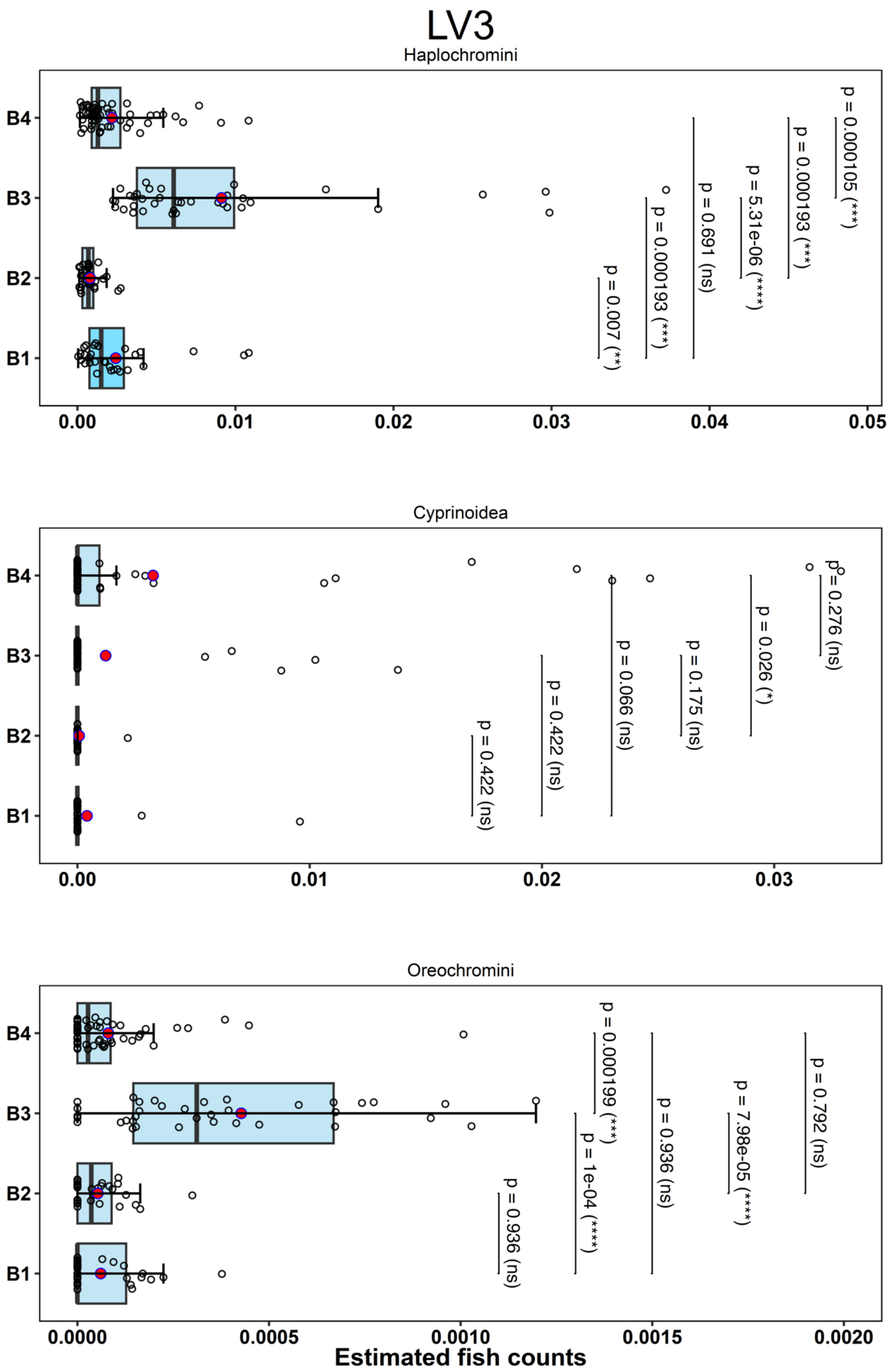


**Supplementary Figure 5d**: Boxplot from the most offshore site, LV3.

# Supplementary references


1. Fryer, G. & Iles, T. D. Cichlid fishes of the great lakes of Africa. (1972).
2. Hamilton, S. E. *et al.* High-resolution bathymetries and shorelines for the Great Lakes of the White Nile basin. *Sci Data* **9**, 642 (2022).
3. Temoltzin-Loranca, Y. *et al.* A chronologically reliable record of 17,000 years of biomass burning in the Lake Victoria area. *Quat. Sci. Rev.* **301**, 107915 (2023).
4. James, N. A. & Matteson, D. S. ecp: An R Package for Nonparametric Multiple Change Point Analysis of Multivariate Data. (2013).
5. Greenwood, P. H. The characin fishes of Lakes Victoria and Kyoga. *Annals and Magazine of Natural History* **2**, 41–47 (1959).
6. Van Oijen, M. J. P. Appendix I. Key to Lake Victoria fishes other than haplochromine cichlids. *Fish stocks and fisheries of Lake Victoria. A handbook for field observations.* 209–300 (1995).
7. Zanata, A. M. & Vari, R. P. The family Alestidae (Ostariophysi, Characiformes): a phylogenetic analysis of a trans-Atlantic clade. *Zool. J. Linn. Soc.* **145**, 1–144 (2005).
8. Corbet, P. S. The food of non-cichlid fishes in the lake Victoria basin, with remarks on their evolution and adaptation to lacustrine conditions. *Proc. Zool. Soc. Lond.* **136**, 1–101 (2009).
9. Gee, J. M. The genus Xenoclarias (Pisces, Siluriformes) in Lake Victoria, with a redescription of Xenoclarias eupogon and notes on the biology of the species. *J. Zool.* **175**, 201–217 (1975).
10. Witte, F., De Winter, W. & Van Densen, W. L. T. Appendix II. Biology of the major fish species of Lake Victoria. *Fish stocks and fisheries of Lake Victoria-A handbook for field observations* (1995).
11. Adriaens, D. & Verraes, W. Ontogeny of the osteocranium in the African catfish, Clarias gariepinus Burchell (1822) (Siluriformes: Clariidae): Ossification sequence as a response to functional demands. *J. Morphol.* **235**, 183–237 (1998).
12. Vigliotta, T. R. A phylogenetic study of the African catfish family Mochokidae (Osteichthyes, Ostariophysi, Siluriformes), with a key to genera. *Proceedings of the Academy of Natural Sciences of Philadelphia* 73–136 (2008).
13. De Vos, L. A systematic revision of the African Schilbeidae (Teleostei, Siluriformes) with an annotated bibliography. *Koninklijk Museum voor Midden-Afrika* (1995).
14. Tweddle, D. *et al.* Occurrence of Barbus profundus Greenwood 1970 (Telostei: Cyprinidae) in mid-water in Lake Victoria. *Afr. J. Aquat. Sci.* **31**, 155–157 (2006).
15. Winfield, I. J. & Nelson, J. S. *Cyprinid Fishes: Systematics, biology and exploitation*. (Springer Science & Business Media, 2012).
16. Kaufman, L. Catastrophic Change in Species-Rich Freshwater Ecosystems. *Bioscience* **42**, 846–858 (1992).
17. Mwanja, M. T. *et al.* Population morphological variation of the Nile perch (Lates niloticus, L. 1758), of East African Lakes and their associated waters. *African Journal of Environmental Science and Technology* **5**, 941–949 (2011).
18. De Vos, L., Snoeks, J. & van den Audenaerde, D. T. An annotated checklist of the fishes of Rwanda (East Central Africa), with historical data on introductions of commercially important species. *J. East Afr. Nat. Hist.* (2001).
19. Trewavas, E. *Tilapiine fishes of the genera Sarotherodon, Oreochromis and Danakilia*. (London, British Museum (Natural History), 1983).
20. Dieleman, J., Van Bocxlaer, B., Nyingi, W. D., Lyaruu, A. & Verschuren, D. Recurrent changes in cichlid dentition linked to climate-driven lake-level fluctuations. *Ecosphere* **10**, e02664 (2019).

21. Greenwood, P. H. Cichlid fishes of Lake Victoria, east Africa : the biology and evolution of a species flock. *Bull. British Mus. Nat. Hist. (Zool.) Suppl.* **6**, 1–134 (1974).
22. Seehausen, O. *Lake Victoria rock cichlids: taxonomy, ecology and distribution*. (Verduyn Cichlids, 1996).
