# Supplementary material for: A continuous fish fossil record reveals key insights into adaptive radiation
Source: Nature. 2023 Oct 4;622(7982):315–20. doi: 10.1038/s41586-023-06603-6 (PMC10567567; doi:10.1038/s41586-023-06603-6)
Supplement: Supplementary file 2 — Reporting Summary [file 41586_2023_6603_MOESM2_ESM.pdf]

nature portfolio

Corresponding author(s): Nare Ngoepe & Ole Seehausen

Last updated by author(s): 7 August 2023

Reporting Summary

Nature Portfolio wishes to improve the reproducibility of the work that we publish. This form provides structure for consistency and transparency in reporting. For further information on Nature Portfolio policies, see our Editorial Policies and the Editorial Policy Checklist.

Statistics

For all statistical analyses, confirm that the following items are present in the figure legend, table legend, main text, or Methods section.

n/a	Confirmed	
☐	☒	The exact sample size (*n*) for each experimental group/condition, given as a discrete number and unit of measurement
☐	☒	A statement on whether measurements were taken from distinct samples or whether the same sample was measured repeatedly
☐	☒	The statistical test(s) used AND whether they are one- or two-sided<br>*Only common tests should be described solely by name; describe more complex techniques in the Methods section.*
☐	☒	A description of all covariates tested
☐	☒	A description of any assumptions or corrections, such as tests of normality and adjustment for multiple comparisons
☐	☒	A full description of the statistical parameters including central tendency (e.g. means) or other basic estimates (e.g. regression coefficient) AND variation (e.g. standard deviation) or associated estimates of uncertainty (e.g. confidence intervals)
☐	☒	For null hypothesis testing, the test statistic (e.g. F, t, r) with confidence intervals, effect sizes, degrees of freedom and P value noted<br>*Give P values as exact values whenever suitable.*
☒	☐	For Bayesian analysis, information on the choice of priors and Markov chain Monte Carlo settings
☐	☒	For hierarchical and complex designs, identification of the appropriate level for tests and full reporting of outcomes
☒	☐	Estimates of effect sizes (e.g. Cohen's *d*, Pearson's *r*), indicating how they were calculated

Our web collection on statistics for biologists contains articles on many of the points above.

Software and code

Policy information about availability of computer code

Data collection	We did not use any software to collect data.
Data analysis	We used Rstudio version 4.3.1 with packages rstatix v0.7.2, ggplot2 v3.4.2, tidypaleo v0.1.3, patchwork 1.1.2, scales v1.2.1, ggtext v0.1.2, rbacon v3.1.1, and dplyr v1.1.2.

For manuscripts utilizing custom algorithms or software that are central to the research but not yet described in published literature, software must be made available to editors and reviewers. We strongly encourage code deposition in a community repository (e.g. GitHub). See the Nature Portfolio guidelines for submitting code & software for further information.

Data

Policy information about availability of data

All manuscripts must include a data availability statement. This statement should provide the following information, where applicable:

- Accession codes, unique identifiers, or web links for publicly available datasets
- A description of any restrictions on data availability
- For clinical datasets or third party data, please ensure that the statement adheres to our policy

All the data generated and analyzed in the current study, including the code to process the data and reproduce all the figures presented here are available on figshare repository.

Research involving human participants, their data, or biological material

Policy information about studies with human participants or human data. See also policy information about sex, gender (identity/presentation), and sexual orientation and race, ethnicity and racism.

Reporting on sex and gender	NA
Reporting on race, ethnicity, or other socially relevant groupings	NA
Population characteristics	NA
Recruitment	NA
Ethics oversight	NA

Note that full information on the approval of the study protocol must also be provided in the manuscript.

Field-specific reporting

Please select the one below that is the best fit for your research. If you are not sure, read the appropriate sections before making your selection.

☐ Life sciences ☐ Behavioural & social sciences ☒ Ecological, evolutionary & environmental sciences

For a reference copy of the document with all sections, see nature.com/documents/nr-reporting-summary-flat.pdf

Life sciences study design

All studies must disclose on these points even when the disclosure is negative.

Sample size	7623 fish teeth fossils
Data exclusions	fossil bones, scales and unassignable fossils
Replication	NA
Randomization	NA
Blinding	NA

Behavioural & social sciences study design

All studies must disclose on these points even when the disclosure is negative.

Study description	NA
Research sample	NA
Sampling strategy	NA
Data collection	NA
Timing	NA
Data exclusions	NA
Non-participation	NA
Randomization	NA

Ecological, evolutionary & environmental sciences study design

All studies must disclose on these points even when the disclosure is negative.

Study description	We used 7623 fish teeth fossils from multiple sites in Lake Victoria to address some major hypothesis at the beginning of an adaptive radiation. We reveal arrival order, relative abundance and habitat occupation of all major fish lineages in the system. We calculated fish estimates from a combination of fish fossil influx for each taxon and the average of the typical number of teeth the modern fish has in the mouth deduced from the published tooth counts of representative species of each major taxon and our reference collection. We reported the fish estimates and detected significant distributional changes through time in the fish composition and abundance of taxa with the multivariate changepoints detection E-divisive method.
Research sample	Multiple representing a wide range of present day fish families found in Lake Victoria were used in the study to interpret the fossil data and assign to taxa. From Alestidae (Brycinus jacksonii), Bagridae (Bagrus docmak), Cichlidae (Astatoreochromis alluaudi, Astatotilapia nubila, Enterochromis paropius, Gaurochromis hiatus, Haplochromis purple yellow, Harpagochromis cf. serranus, Labrochromis stone, Lipochromis melanopterus, Lithochromis sp. (scraper pseudonigricans), Lithochromis sp. yellow chin pseudonigricans, Mbipia lutea, Mbipia mbipi, Neochromis gigas, Neochromis omniceruleus, Neochromis rufocaudalis, Neochromis sp. (uniscupid scraper), Paralabidochromis chilotes, Paralabidochromis cyaneus, Paralabidochromis flavus, Paralabidochromis sp. rockkribensis, Paralabidochromis sp. (short snout scraper), Platytaeniodus degeni, Psammochromis riponianus, Ptyochromis sauvagei or P. fisheri, Ptyochromis xenognathus, Pundamilia macrocephala, Pundamilia nyererei, Pundamilia pundamilia, Pundamilia sp. (pink anal), Yssichromis laparogramma, Yssichromis pyrrhocephalus), Clariidae (Clarias sp.), Cypriniformes (Rastrineobola argentea, Labeo victorianus, Enteromius sp.), Latidae (Lates niloticus), Mochokidae (Synodontis victoriae), Mastacembelidae (Mastacembelus frenatus), and Oreochromini (Oreochromis variabilis, Oreochromis esculentus, Oreochromis niloticus, and Oreochromis leucosticus). And the sediment core dates and volumes are from<br>Temoltzin-Loranca et al. 2023.
Sampling strategy	The sediment cores were collected in 2018 from four sites; LVC18-S1 (located at 01°06,914′ S, 33°55,146′ E), LVC18-S2 (located at 01°07,850′ S, 33°56,780′ E), LVC18-S3 (located at 01°06,914′ S, 33°55,146′ E) and LVC18-S4 (located at 01°02,966′ S, 33°47,768′ E). We refer to the sites as LV1, LV2, LV3 and LV4, respectively. The cores were collected along a transect of increasing water depths (LV3 at 13 m, LV2 at 22 m, LV1 at 37 m, LV4 at 63 m) and distance from shore (LV3 at 2 km, LV2 at 6 km, LV1 at 9 km, LV4 at 30 km from the shore) in the Shirati Bay area of Lake Victoria.
Data collection	The sediment cores were taken by a team led by Moritz Muschick, Mary Kishe and Salome Mwaiko, using an UWITEC platform with metal floats, which was transported to the sites by TAFIRI's RV Lake Victoria Explorer and positioned using four large steel anchors. A Niederreiter-type piston-corer with a 3m drive length, 60mm liner diameter, motorized hammer and hydraulic core catcher was suspended from the platform. Parallel holes with contiguous drives were cored in sites LV1, LV2 and LV3, and a series of overlapping drives from adjacent holes in LV4. Cores were cut into ~1m long sections, which were sealed and labelled and kept cool during transport and storage. Yunuen Temoltzin-Loranca sub-sampled the cores contiguously at 2 cm intervals, and the samples were then wet-sieved through stacked 200 µm and 100µm mesh-size sieves to retain fish bones, scales and teeth. Nare Ngoepe screened the<br>3nature portfolio | reporting summary March 2021<br>samples with ZEISS stereo-microscope Stemi 508 at 10x magnification (Carl Zeiss, Heidelberg, Germany) sorted the subsamples for fossils. The recovered fossils were individually photographed and a detailed record with images and notes was created. Fossils were then visually analyzed and compared with literature and our reference collection to assign each fossil to a taxonomic group.
Timing and spatial scale	The sediments cores were collected from 10.10.2018 to 31.10.2018. The sub-sampling, fossil screening, fossil sorting and taxa assignments were continuous from June 2019 to December 2022.
Data exclusions	Fossil bones and scales were excluded as they cannot be morphologically assigned to a taxon, that information we could only obtain from fish teeth fossils.
Reproducibility	All the calculations on our data can be reproduced. The study design is also reproduceable and we provided a detailed reference images of teeth that can also be used.
Randomization	We had digital photograph catalogue of modern tooth specimens of Lake Victoria fishes that we used as a reference to assign to different taxa. The collection comprised 40 species representing seven families that form the modern assemblage in Lake Victoria. We also used published descriptions of teeth together with images and drawings to assign the fossil data into taxa.
Blinding	No data blinding was performed, all the fish taxon had unique teeth morphologies.

Did the study involve field work? ☒ Yes ☐ No

Field work, collection and transport

Field conditions	Core collection was undertaken in October 2018 on the central western shore of Lake Victoria at the locations given below. Calm winds in the early morning hours allowed coring from an anchored platform in both shallow and deep waters. The joint expedition of the University of Bern and the Tanzania Fisheries Research Institute (TAFIRI) was carried out using the modular UWITEC coring platform of the Institute of Plant Sciences of the University of Bern and TAFIRI's research vessel Lake Victoria Explorer.
Location	Tanzania, Lake Victoria, Shirati Bay, Four sites; LVC18-S1 (located at 01°06,914′ S, 33°55,146′ E), LVC18-S2 (located at 01°07,850′ S, 33°56,780′ E), LVC18-S3 (located at 01°06,914′ S, 33°55,146′ E) and LVC18-S4 (located at 01°02,966′ S, 33°47,768′ E).

Access & import/export	Coring was undertaken with permission of COSTECH under research permit No. 2018-237-NA-2018-57. The coring platform and ancillary research equipment was temporarily imported and samples exported with permission of the Ministry of Livestock and Fisheries, United Republic of Tanzania.
Disturbance	Assembly, transport and positioning of the platform, and coring of 73.8 m (total) of sediment cores caused only very localised disturbance to the sediment surface. No tracer fluids were used.

Reporting for specific materials, systems and methods

We require information from authors about some types of materials, experimental systems and methods used in many studies. Here, indicate whether each material, system or method listed is relevant to your study. If you are not sure if a list item applies to your research, read the appropriate section before selecting a response.

Materials & experimental systems

n/a	Involved in the study
☒	☐ Antibodies
☒	☐ Eukaryotic cell lines
☒	☐ Palaeontology and archaeology
☒	☐ Animals and other organisms
☒	☐ Clinical data
☒	☐ Dual use research of concern
☒	☐ Plants

Methods

n/a	Involved in the study
☒	☐ ChIP-seq
☒	☐ Flow cytometry
☒	☐ MRI-based neuroimaging

Antibodies

Antibodies used	NA
Validation	NA

Eukaryotic cell lines

Policy information about cell lines and Sex and Gender in Research

Cell line source(s)	NA
Authentication	NA
Mycoplasma contamination	NA
Commonly misidentified lines (See ICLAC register)	NA

Palaeontology and Archaeology

Specimen provenance	NA
Specimen deposition	NA
Dating methods	NA

☐ Tick this box to confirm that the raw and calibrated dates are available in the paper or in Supplementary Information.

Ethics oversight	NA

Note that full information on the approval of the study protocol must also be provided in the manuscript.

Animals and other research organisms

Policy information about studies involving animals; ARRIVE guidelines recommended for reporting animal research, and Sex and Gender in Research

Laboratory animals	NA

Wild animals	NA
Reporting on sex	NA
Field-collected samples	NA
Ethics oversight	NA

Note that full information on the approval of the study protocol must also be provided in the manuscript.

Clinical data

Policy information about clinical studies

All manuscripts should comply with the ICMJE guidelines for publication of clinical research and a completed CONSORT checklist must be included with all submissions.

Clinical trial registration	NA
Study protocol	NA
Data collection	NA
Outcomes	NA

Dual use research of concern

Policy information about dual use research of concern

Hazards

Could the accidental, deliberate or reckless misuse of agents or technologies generated in the work, or the application of information presented in the manuscript, pose a threat to:

No	Yes	
☒	☐	Public health
☒	☐	National security
☒	☐	Crops and/or livestock
☒	☐	Ecosystems
☒	☐	Any other significant area

Experiments of concern

Does the work involve any of these experiments of concern:

No	Yes	
☒	☐	Demonstrate how to render a vaccine ineffective
☒	☐	Confer resistance to therapeutically useful antibiotics or antiviral agents
☒	☐	Enhance the virulence of a pathogen or render a nonpathogen virulent
☒	☐	Increase transmissibility of a pathogen
☒	☐	Alter the host range of a pathogen
☒	☐	Enable evasion of diagnostic/detection modalities
☒	☐	Enable the weaponization of a biological agent or toxin
☒	☐	Any other potentially harmful combination of experiments and agents

Plants

Seed stocks	NA
Novel plant genotypes	NA
Authentication	NA

ChIP-seq

Data deposition

☐ Confirm that both raw and final processed data have been deposited in a public database such as GEO.

☐ Confirm that you have deposited or provided access to graph files (e.g. BED files) for the called peaks.

Data access links<br>*May remain private before publication.*	NA
Files in database submission	NA
Genome browser session<br>(e.g. UCSC)	NA

Methodology

Replicates	NA
Sequencing depth	NA
Antibodies	NA
Peak calling parameters	NA
Data quality	NA
Software	NA

Flow Cytometry

Plots

Confirm that:

☐ The axis labels state the marker and fluorochrome used (e.g. CD4-FITC).

☐ The axis scales are clearly visible. Include numbers along axes only for bottom left plot of group (a 'group' is an analysis of identical markers).

☐ All plots are contour plots with outliers or pseudocolor plots.

☐ A numerical value for number of cells or percentage (with statistics) is provided.

Methodology

Sample preparation	NA
Instrument	NA
Software	NA
Cell population abundance	NA
Gating strategy	NA

☐ Tick this box to confirm that a figure exemplifying the gating strategy is provided in the Supplementary Information.

Magnetic resonance imaging

Experimental design

Design type	NA
Design specifications	NA
Behavioral performance measures	NA

Acquisition

Imaging type(s)	NA
Field strength	NA
Sequence & imaging parameters	NA
Area of acquisition	NA

Diffusion MRI ☐ Used ☐ Not used

Preprocessing

Preprocessing software	NA
Normalization	NA
Normalization template	NA
Noise and artifact removal	NA
Volume censoring	NA

Statistical modeling & inference

Model type and settings	NA
Effect(s) tested	NA

Specify type of analysis: ☐ Whole brain ☐ ROI-based ☐ Both

Statistic type for inference (See Eklund et al. 2016)	NA
Correction	NA

Models & analysis

n/a	Involved in the study
☒	☐ Functional and/or effective connectivity
☒	☐ Graph analysis
☒	☐ Multivariate modeling or predictive analysis

Functional and/or effective connectivity	NA
Graph analysis	NA
Multivariate modeling and predictive analysis	NA
